# Subgingival microbiome dynamic alteration associated with necrotizing periodontal disease: A case report

**DOI:** 10.1097/MD.0000000000024311

**Published:** 2021-02-26

**Authors:** Jia Jia, You Zhou, Xinwen Wang, Yuan Liu

**Affiliations:** a Lanzhou Hospital of Stomatology, Lanzhou, Gansu Province; b Department of Oral Medicine; c Department of Preventive Dentistry, School of Stomatology, The Fourth Military Medical University; d Shaanxi Clinical Research Center for Oral Diseases, National Clinical Research Center for Oral Diseases, State Key Laboratory of Military Stomatology; e Department of Oral Histology and Pathology, School of Stomatology, The Fourth Military Medical University, Xi’an, Shaanxi Province, China.

**Keywords:** *actinobacteria*, *corynebacterium*, *fusobacteria*, *leptotrichia*, necrotizing periodontal diseases, necrotizing ulcerative periodontitis, *neisseria*

## Abstract

**Rationale:**

Necrotizing periodontal diseases (NPDs) are a group of infectious diseases varying in severity, and microorganisms are responsible for these diseases. Currently, the oral microbiota in early disease has been poorly investigated; thus, the causative pathogen and dynamic alteration of the microbiome in NPDs remain unclear.

**Patient concerns:**

We report a case of a 33-year-old female patient with severe gingival pain and localized necrotizing ulcerative gingival lesions. Conventional therapy was performed, but the necrotizing lesion continued to develop.

**Diagnoses:**

X-ray examination showed marginal alveolar bone loss in the involved teeth. Histological examination of a biopsy from the gingival lesion showed chronic inflammatory cell infiltration in the tissue, and no cancer cells were observed. Subgingival swabs were taken from the ulcerative gingiva and the gingiva that was not yet affected, and the composition of the microbiota was analyzed by targeted pyrosequencing of the V3-V4 hypervariable regions of the small subunit ribosomal RNA. We found that *Neisseria* spp., *Corynebacterium* spp., and *Prevotella* spp. were clearly enriched in the lesion site. However, *Fusobacteria* was more abundant in the not-yet-affected gingiva, and *Leptotrichia* spp. were the most abundant phylotype.

**Interventions:**

After clinical assessment, a tooth with poor prognosis was extracted, and minocycline hydrochloride was locally administered in the involved tooth pocket every day. Additionally, the patient received 100 mg of hydrochloric acid doxycycline twice per day.

**Outcomes:**

Remarkable improvement was obtained after 3 days, and the lesion completely healed after 1 week. The follow-up examination 1 year later showed a complete recovery with no recurrent episodes of pain.

**Lessons:**

Changes in the subgingival microbiome can occurr before clinical symptoms appears, and *Fusobacteria* may be involved in the imbalance of the subgingival flora in the early stage of NPDs. Moreover, *Neisseria* is a potential bacterial candidate that deserves further study.

## 1 Introduction

Necrotizing periodontal diseases (NPDs) are a group of infectious diseases with similar etiologies, clinical characteristics and treatments. They occur mainly in sub-Saharan Africa, Latin America, and Asia^[1]^ and may present as necrotizing ulcerative gingivitis (NUG), necrotizing ulcerative periodontitis (NUP), or necrotizing stomatitis and vary in disease severity.^[2,3]^ NPD develops rapidly. Often, the necrotizing lesion extends laterally from the papilla to the gingival margin and progresses to other sites in the mouth, evolving from a localized disease into a generalized disease and causing severe tissue destruction. Thus, NPDs are among the most severe inflammatory conditions associated with oral biofilm bacteria.

Although certain types of microorganisms including *Prevotella, Actinomyces* and *Fusobacteria* have been identified in lesions,^[4–6]^ scholars suspect that the microbiological findings may reflect just the development or result of the disease rather than the real etiology. Currently, the oral microbiota in early cases has been poorly investigated. Thus, the causative pathogen and dynamic alteration of the microbiome in NPDs remain unclear, hampering effective prevention and treatment of NPDs. Here, we present a case of localized NUP, where the subgingival bacterial composition of NUP sites and the site not yet affected were analyzed, possibly providing clues regarding the dynamic alteration of microbiome in NPDs.

## 2 Case report

A 33-year-old female presented to the oral medicine service with a chief complaint of severe gingival pain and lesions for 1 month. The patient was a nonsmoker and had no significant medical history or known allergies.

**Figure 1:**
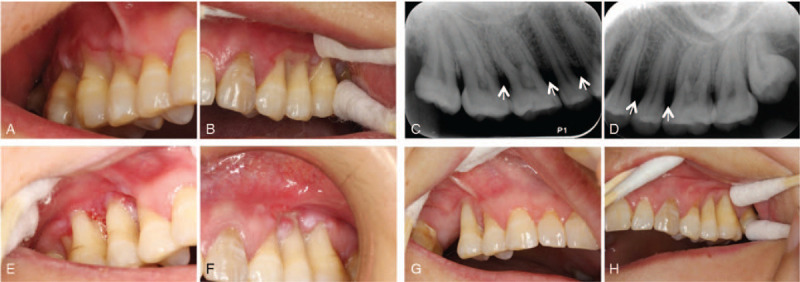
Clinical and intraoral periapical radiographs. (a,b) Initial clinical views of affected teeth. (c,d) Initial periapical radiographs of affected teeth showing alveolar bone loss of premolars and molars. (e,f) Clinical presentation at 5 months after traditional treatment. (g,h) Clinical presentation at 1 year after tetracycline treatment.

The clinical examination revealed erythematous and ulcerative gingiva localized at the buccal side from teeth 15 to 17 and 24 to 27 (Federation Dentaire Internationale tooth numbering system), and oral hygiene was good (Fig. 1a, b). X-ray examination showed marginal alveolar bone loss in the involved teeth, and periodontal resorption of alveolar bone in teeth 15, 16, 25, and 26 was obvious (Fig. 1c, d). The laboratory tests performed included a complete blood count, autoantibody detection, syphilis serology and anti-HIV antibody detection, and the results were all normal. The diagnosis of necrotizing ulcerative periodontitis (NUP) was made.

Ten volumes of diluted hydrogen peroxide were administered to the necrotic lesions with a sterile cotton roll in conjunction with topical administration of metronidazole every other day. The patient was prescribed an oral mouth rinse (0.2% chlorhexidine twice daily for 5 days). Five days later, the clinical examination showed an improvement in symptoms, with resolution of the ulcerated pseudomembranous area, and then scaling and root planing with local anesthesia was performed. The patient showed obvious relief of pain. However, 10 days later, the condition relapsed. In addition to the therapy above, the patient received 500 mg of amoxicillin twice per day for 5 days, but the lesions did not completely heal, and she made no further visits.

Five months later, the patient presented to the department with a complaint of aggravated pain of the gingiva and movable teeth. The clinical examination revealed ulcerative and obviously recessed gingiva (Fig. 1e, f), and teeth 15, 16, 25, and 26 had different degrees of mobility. To further confirm the diagnosis, after obtaining informed consent from the patient, a biopsy was taken from the ulcerative gingiva. Microbial samples were taken from the subgingival plaque of teeth in the ulcerative (NUP)/not-yet-affected (NUP-n) region. NUP samples were collected from teeth 16 and 26, and NUP-n samples were collected from 18 and 28. Bacterial DNA was extracted using the CTAB/SDS method. 16S rRNA genes of V3-V4 region were amplified with the barcode. All PCRs were performed with Phusion High-Fidelity PCR Master Mix (New England Biolabs). After purification of the PCR products, sequencing libraries were generated using a TruSeq DNA PCR-free Sample Preparation Kit (IL) following the manufacturer's recommendations, and index codes were added. The library was sequenced on an IlluminaHiSeq2500 platform, and 250-bp paired-end reads were generated. Sequences analysis was performed using Uparse software (Uparse v 7.0.1001). For each representative sequence, the Silva Database (http://www.arb-silva.de/) was used based on the RDP classifier algorithm to annotate taxonomic information.

**Figure 2:**
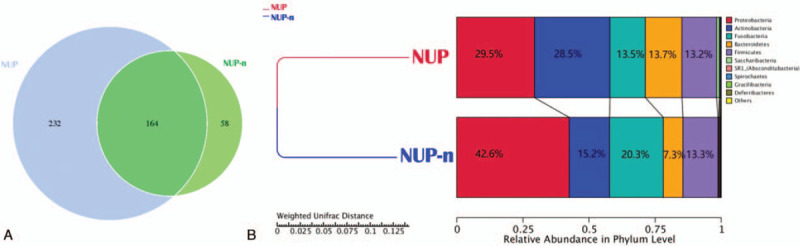
Taxonomic analysis of the subgingival microbiota. (a) Venn diagram showing the unique and shared OTUs between the NUP and control sample. (b) Relative abundance of phyla in each sample from 16S rRNA gene sequencing.

Hematoxylin and eosin (H&E)-stained tissue showed chronic inflammatory cell infiltration in the tissue, and no cancer cells were observed. Through the 16S rRNA sequencing analysis, a total of 396 and 222 operational taxonomic units (OTUs) were found in the NUP and NUP-n samples, respectively (Fig. 2a), and they were assigned at the phylum and genus taxonomic level (19 bacterial phyla, 185 genera), providing a comparative review of the bacterial diversity and composition among them (Fig. 2b, Table 1).

**Table 1:**
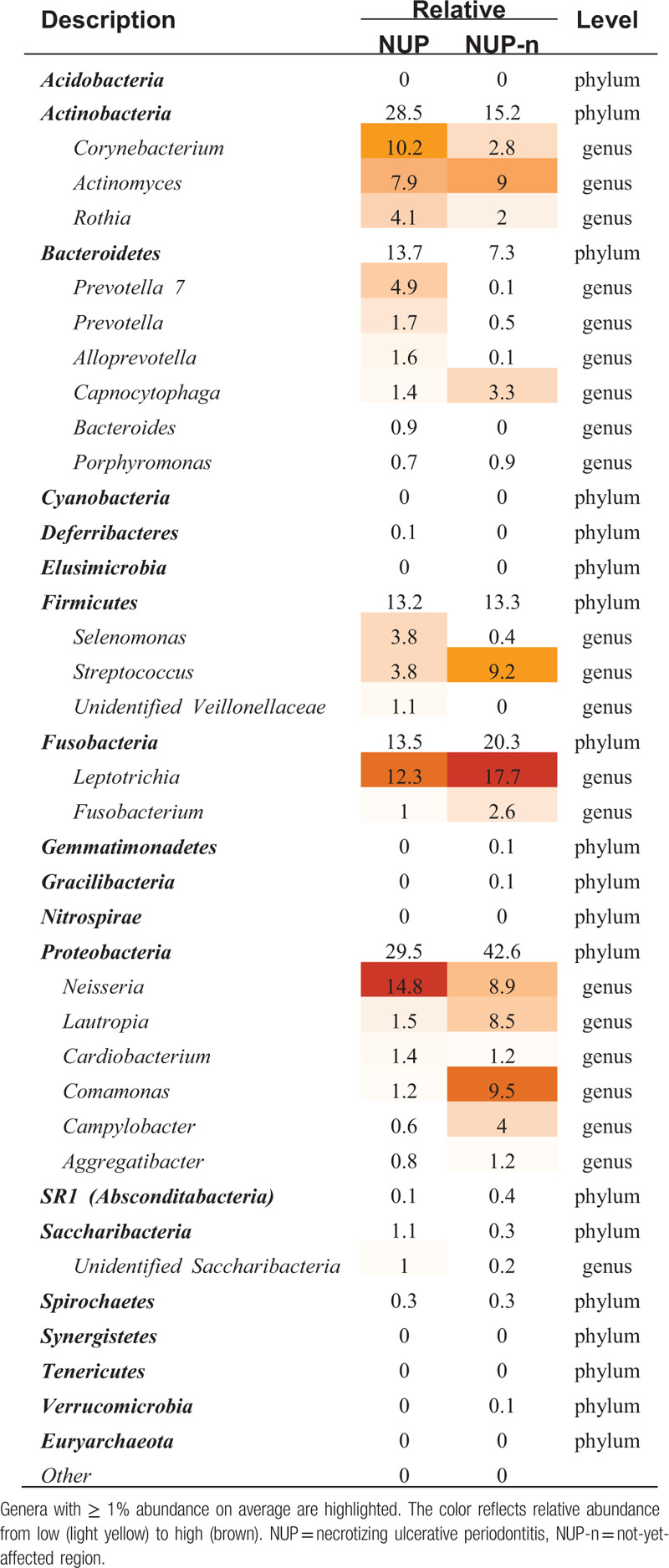
Distribution of subgingival bacteria at the phylum and genus (top 20) level in each group.

In the NUP sample, *Proteobacteria* (29.5%) and *Actinobacteria* (28.5%) predominated, followed by *Bacteroidetes* (13.7%) and *Fusobacteria* (13.5%). In the NUP-n sample, *Proteobacteria* was the most prevalent phylum (42.6%), followed by *Fusobacteria* (20.3%). The relative abundance of each genus was compared between the groups (Table 1). *Leptotrichia* (phylum *Fusobacteria*) was the most prevalent genus in the NUP-n samples, and its abundance decreased in the NUP sample, along with that of *Comamonas* spp.*, Lautropia* spp.*, Streptococcus, Fusobacterium* and *Actinomyces*. Furthermore, the abundances of *Neisseria* spp.*, Corynebacterium* spp. and *Prevotella* spp. were obviously increased with the development of NUP, and *Neisseria* was the most prevalent genus at the NUP site.

After clinical assessment, tooth 16 was extracted, and minocycline hydrochloride was locally administered in the involved tooth pocket every day. At the same time, the patient received 100 mg of hydrochloric acid doxycycline twice per day. Remarkable improvement was obtained after 3 days, the size and congestion of the necrotized gingiva decreased, and the patient reported being in less pain. The lesion healed after 1 week, and the gingiva had epithelialized completely and had an almost normal appearance. The follow-up examination 1 year later showed recovery with no mobility of teeth or recurrent episodes of pain despite the exposed root surfaces being still visible (Fig. 1g, h).

Written informed consent was obtained from the patient for the publication of this case report and its accompanying images.

## 3 Discussion

In this case, we observed a higher diversity of bacteria in lesion sites (396 OTUs) than in the NUP-n sites (222 OTUs), which is consistent with previous reports showing that bacterial microbiota diversity is higher in diseased patients than in healthy patients.^[7,8]^ A total of 19 bacterial phyla (185 genera) were detected based on the sequence analysis. Many abundant species or phylotypes, including *Prevotella* spp. and *Actinobacteria* members such as *Corynebacterium, Actinomyces* spp. and *Rothia*, were found in the lesion sites that have been previously associated with periodontitis,^[7,9]^ further confirming that these bacteria are essential to the development of NPDs.

The role of *Fusobacteria* in NUPs has been controversial. In 1999, Falkler et al detected *Fusobacteria* in most noma patients^[4,10]^ but in only 1 healthy person and hypothesized that it could be responsible for the onset of NUPs. However, in the current largest microbiological study on NPDs performed by the Geneva Study Group on Noma (GESNOMA) project, *Fusobacteria,* such as *Leptotrichia* and *Fusobacterium,* were less abundant in lesions than in healthy control tissues, raising doubt that this species is associated with NPD lesions.^[11]^ In 2002, Paster et al detected bacterial species in advanced NUP lesions using a culture-independent molecular method, and the results showed a notable absence of *Fusobacterium* spp., which are usually expected in these infections.^[12]^ In the present study, we found that *Fusobacteria* abundance was decreased in lesion sites, which was in agreement with the latter report. However, in the NUP-n gingiva of this patient, a member of *Fusobacteria, Leptotrichia* spp., was the most abundant phylotype instead of *Streptococcus*, which is substantially different from the microbiota of healthy gingiva described in the existing literature.^[7,13,14]^

According to the studies associated with periodontally healthy populations from different regions and races, *Firmicutes*, especially *Streptococcus,* is consistently the most abundant bacteria in the subgingival microbiota.^[7,13–16]^ Previously, very little research had addressed the microbiota of not-yet-affected gingiva in NPD patients. In the GESNOMA project, samples from the not-yet-affected sites of NPD patients were enrolled in healthy site libraries,^[11]^ and the researchers obtained a negative result and concluded that *Fusobacteria* was not associated with NPD. However, the high presentation of *Fusobacteria* in the NUP-n gingiva of this patient suggested that subgingival microbiome changes may have occurred before clinical symptoms appeared. *Fusobacteria* might be involved in the imbalance of the subgingival flora in the early stage of NPD. In addition, *Comamonas* spp., *Lautropia* spp., and *Actinomyces*, which were previously reported to be involved in periodontal disease,^[17–19]^ were also abundant in NUP-n sites but less prevalent in lesion gingiva, and they might play a role similar to that of *Fusobacteria*.

The role of *Neisseria* in NPD has attracted limited attention, and the few published studies related to NPD showed changes in this phylotype. In an oral microbiome study on HIV-associated periodontitis, *Neisseria* was the only genus that was consistently enriched in HIV-positive participants regardless of the periodontal probing depth, highlighting a possible role of *Neisseria* in HIV-associated oral pathogenesis.^[20]^ Interestingly, HIV-infected patients are currently the main population suffering from NUP.^[21]^ The present study showed that *Neisseria* was an abundant bacterial genus in NUP-n sites; moreover, *Neisseria* abundance increased continuously in NUP lesions, highlighting that the roles of *Neisseria* in the development of NUP should not be neglected and deserve further study.

The conventional treatment of NUG, consisting of superficial debridement, oral hygiene instruction and prescription of mouthwash, aims to reduce microbial dental plaque and eliminate acute inflammation.^[3,22,23]^ If patients have systemic symptoms related to NUG, antibiotics are recommended.^[23]^ Metronidazole is a common first-line drug choice due to its activity against anaerobes.^[24]^ However, sometimes metronidazole administration has a minor impact.^[25]^ For refractory subjects, in addition to periodontal scaling and treatment with “classic” oral antibiotics, intervention must be tailored to the individual's needs.

## Author contributions

**Data curation:** Jia Jia, You Zhou.

**Formal analysis:** Xinwen Wang, Yuan Liu.

**Project administration:** Xinwen Wang.

**Resources:** Jia Jia.

**Supervision:** Xinwen Wang, Yuan Liu.

**Writing – original draft:** Jia Jia, You Zhou.
